# Supplementary figures and images for: A frameshift in Yersinia pestis rcsD alters canonical Rcs signalling to preserve flea-mammal plague transmission cycles
Source: eLife. 2023 Apr 3;12:e83946. doi: 10.7554/eLife.83946 (PMC10191623; doi:10.7554/eLife.83946)

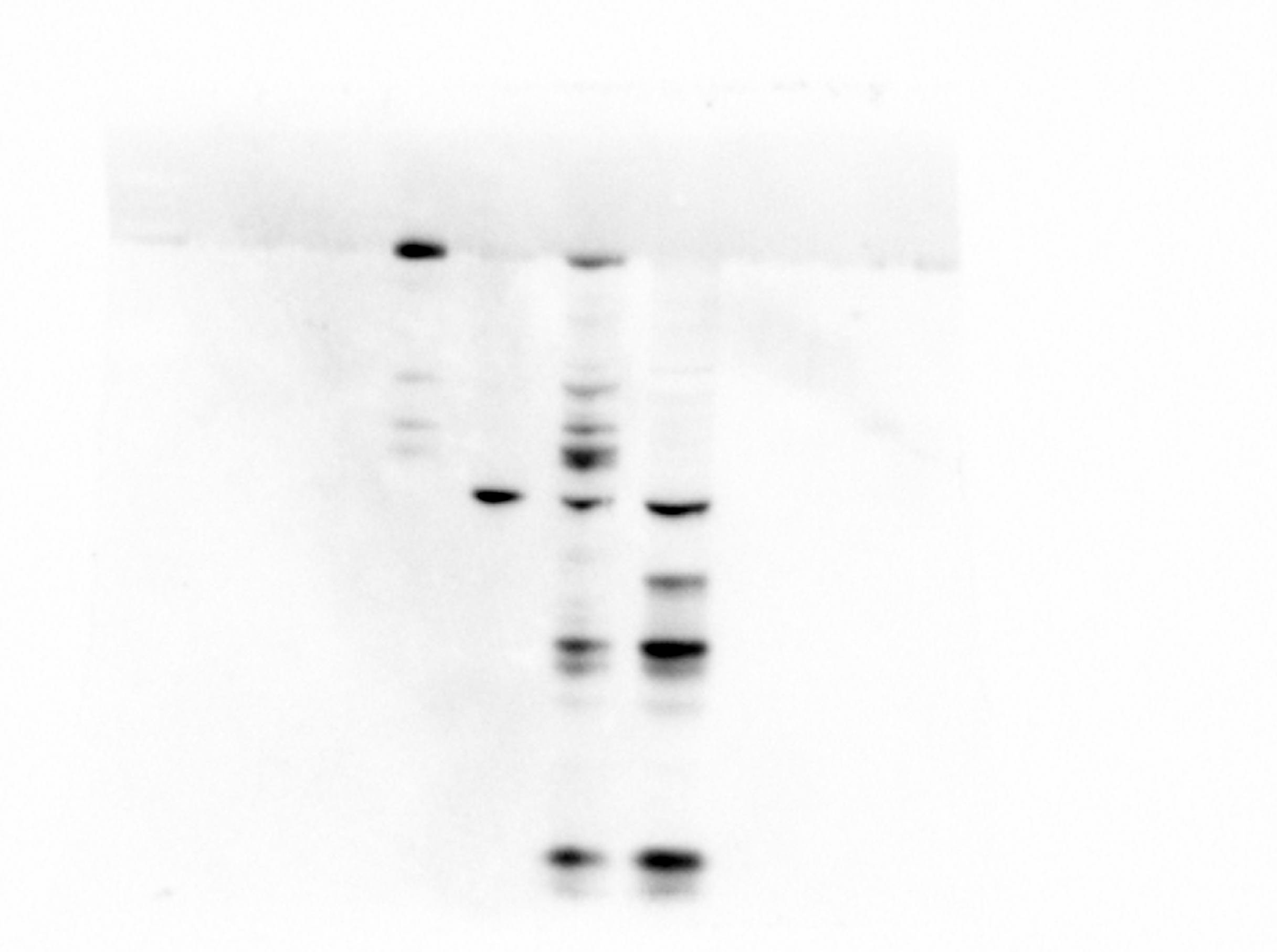

Supplement: Figure 2—source data 1. — The original file of the full raw unedited blots of Figure 2D and the uncropped blot with the relevant bands clearly labelled as Figure 2D. [file elife-83946-fig2-data1.zip › Figure 2D-source data 1-orignal.tif]

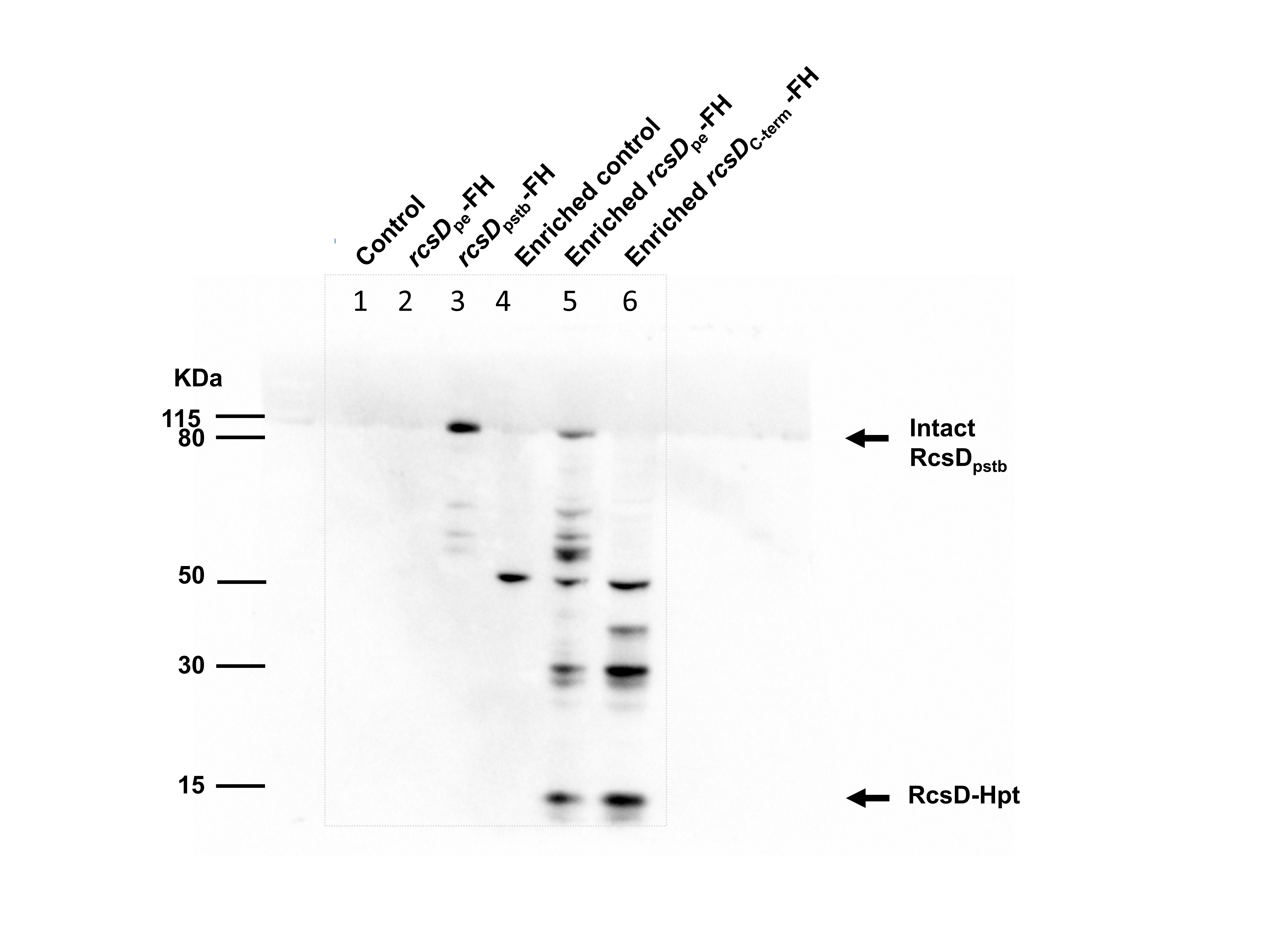

Supplement: Figure 2—source data 1. — The original file of the full raw unedited blots of Figure 2D and the uncropped blot with the relevant bands clearly labelled as Figure 2D. [file elife-83946-fig2-data1.zip › Figure 2D-source data 2-labelled.TIF]

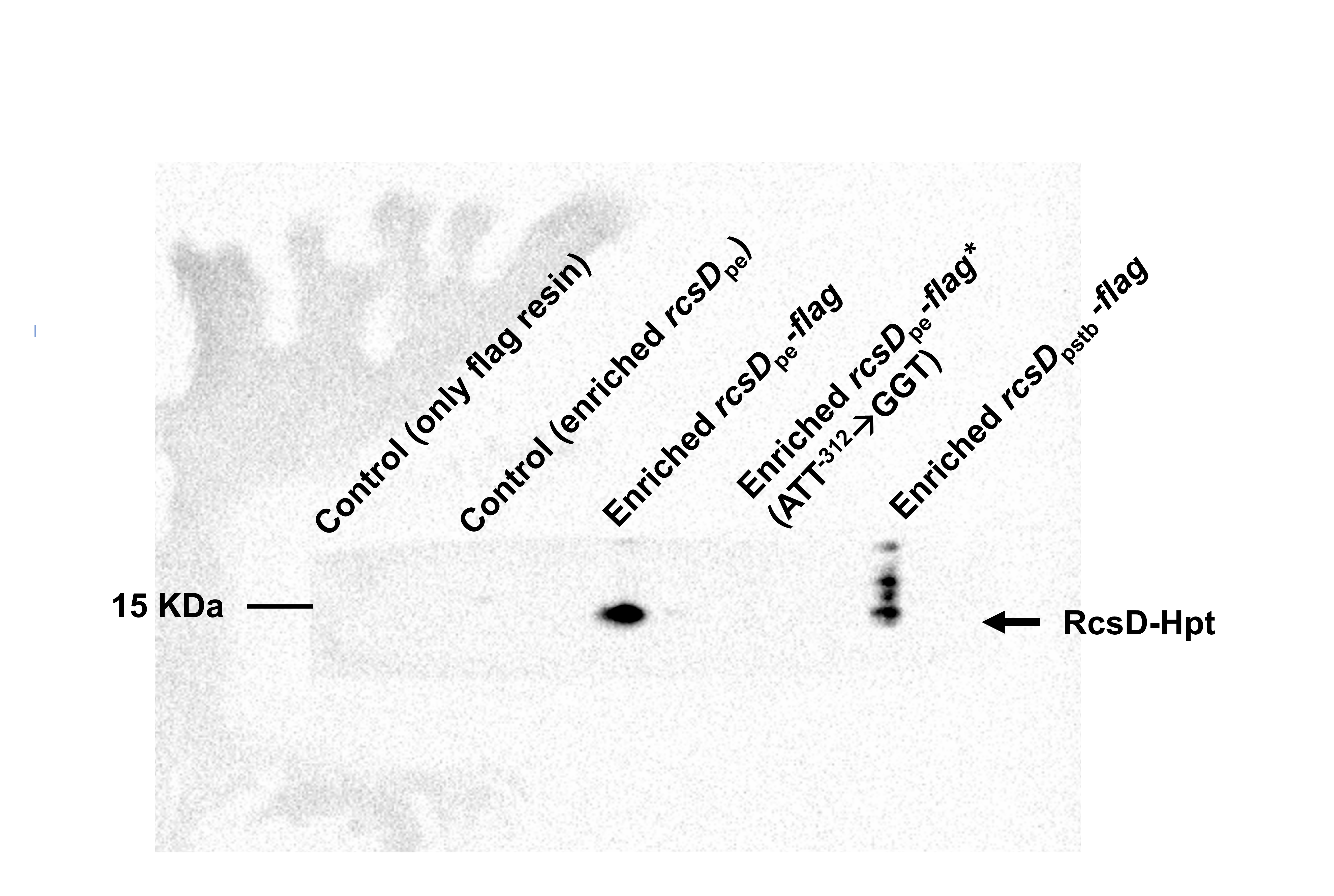

Supplement: Figure 2—figure supplement 1—source data 1. — The original file of the full raw unedited blots and the uncropped blot with the relevant bands clearly labelled as Figure 2—figure supplement 1E. [file elife-83946-fig2-figsupp1-data1.zip › Figure 2-figure supp 1E-source data 1/Figure 2-figure supp 1E-source data 1-labelled.TIF]

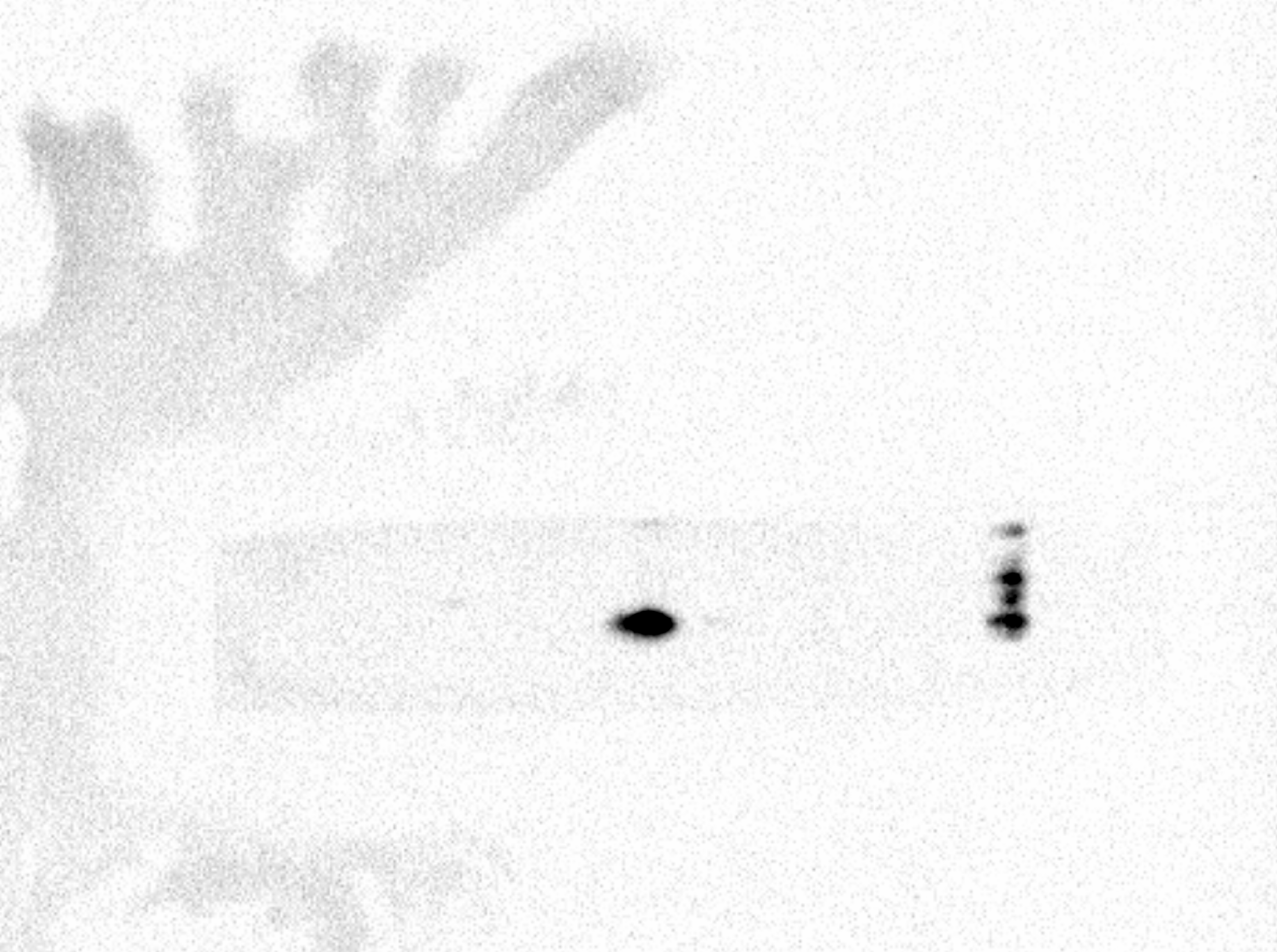

Supplement: Figure 2—figure supplement 1—source data 1. — The original file of the full raw unedited blots and the uncropped blot with the relevant bands clearly labelled as Figure 2—figure supplement 1E. [file elife-83946-fig2-figsupp1-data1.zip › Figure 2-figure supp 1E-source data 1/Figure 2-figure supp 1E-source data 1-original.tif]

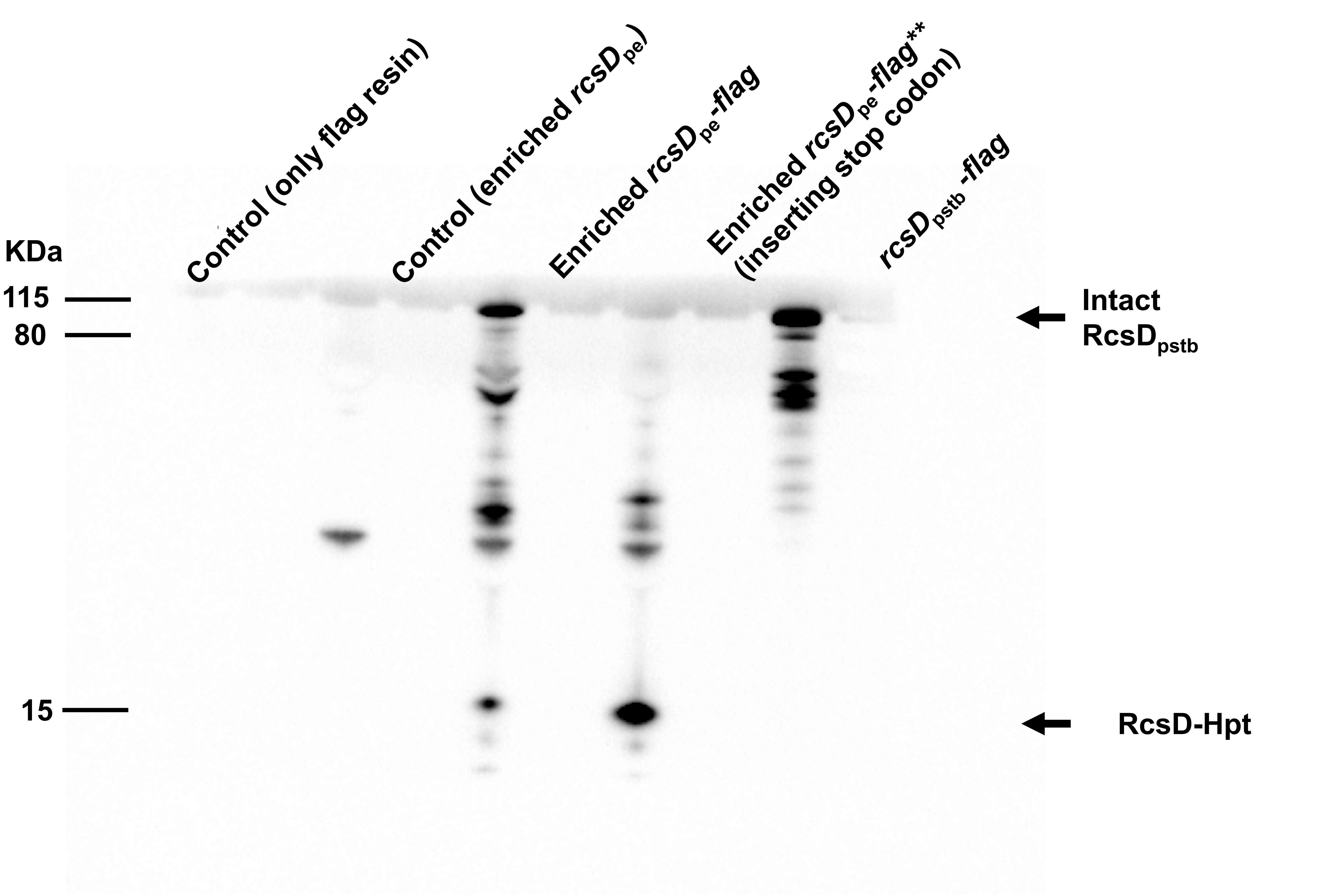

Supplement: Figure 2—figure supplement 1—source data 2. — The original file of the full raw unedited blots and the uncropped blot with the relevant bands clearly labelled as Figure 2—figure supplement 1F. [file elife-83946-fig2-figsupp1-data2.zip › Figure 2-figure supp 1F-source data 2/Figure 2-figure supp 1F-source data 2-labelled.TIF]

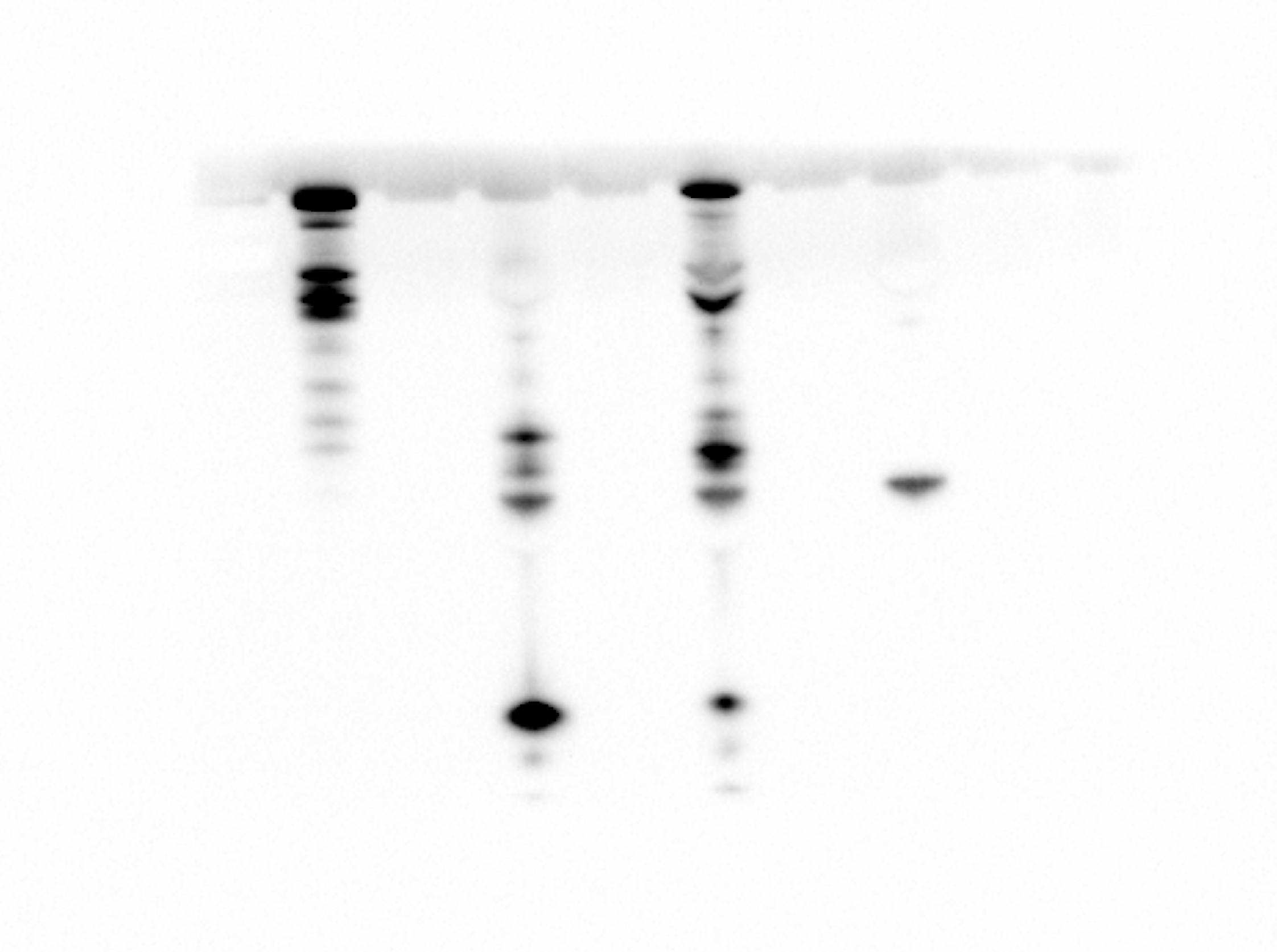

Supplement: Figure 2—figure supplement 1—source data 2. — The original file of the full raw unedited blots and the uncropped blot with the relevant bands clearly labelled as Figure 2—figure supplement 1F. [file elife-83946-fig2-figsupp1-data2.zip › Figure 2-figure supp 1F-source data 2/Figure 2-figure supp 1F-source data 2-orignal.tif]

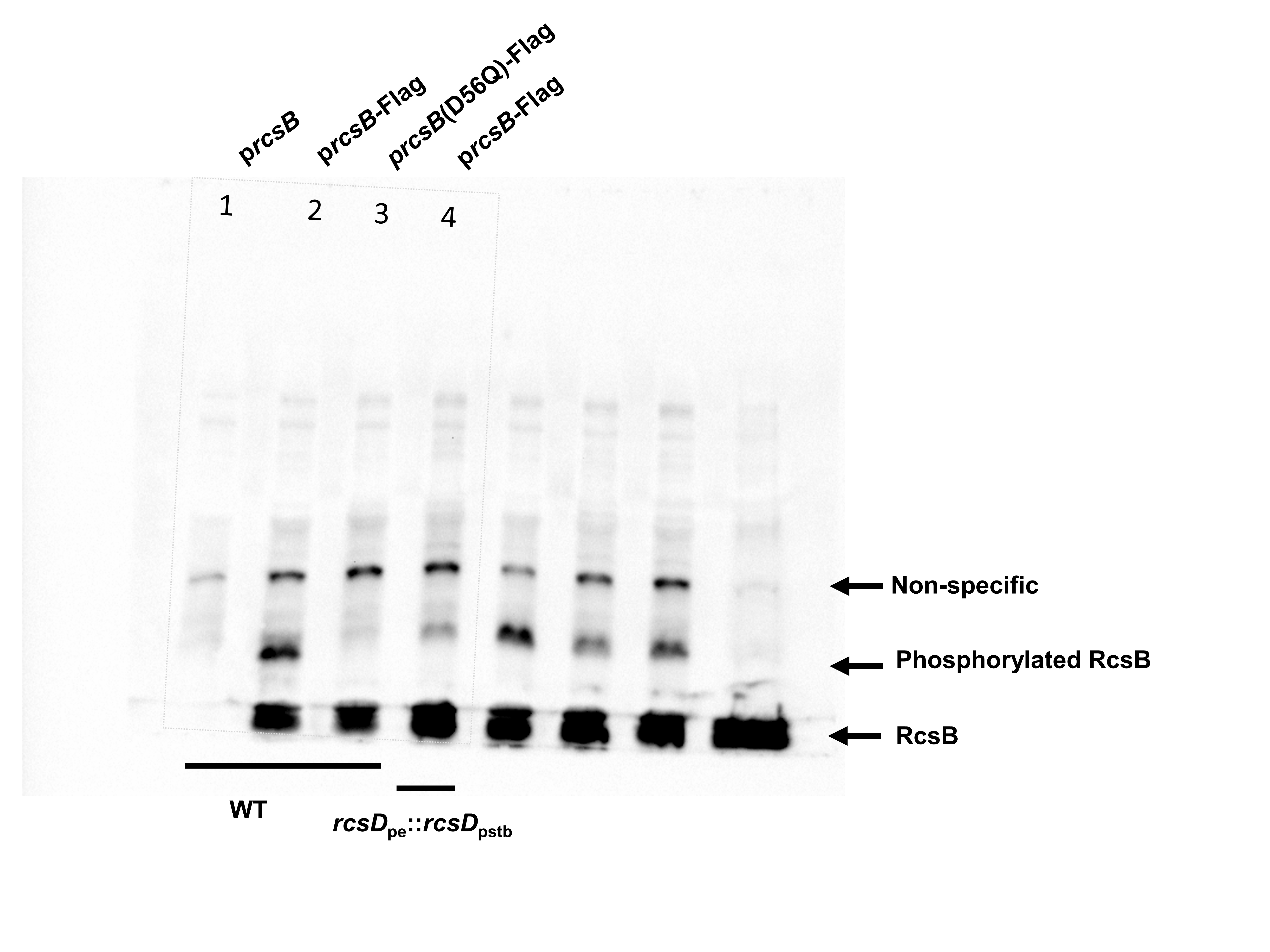

Supplement: Figure 4—source data 1. — The original file of the full raw unedited blots and the uncropped blot with the relevant bands clearly labelled as Figure 4B. [file elife-83946-fig4-data1.zip › Figure 4B-source data 1-labelled.TIF]

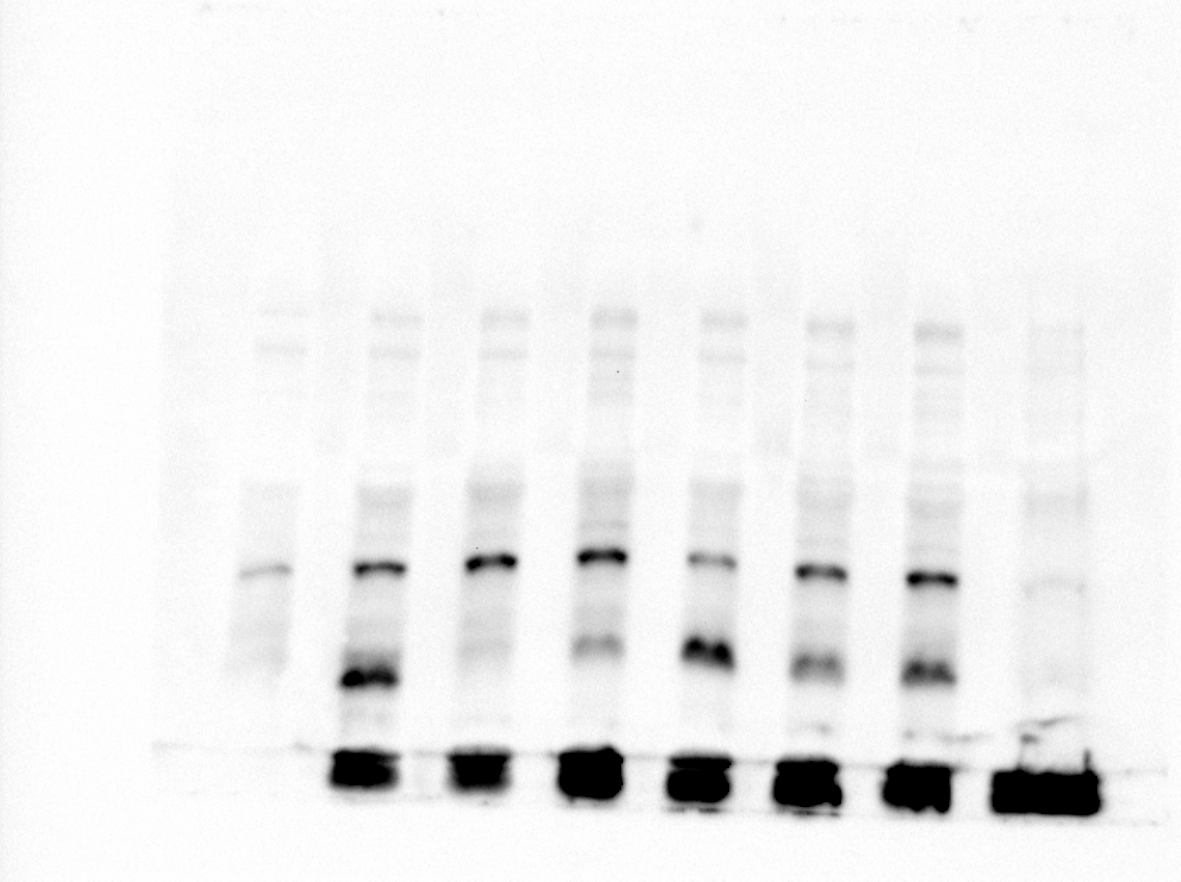

Supplement: Figure 4—source data 1. — The original file of the full raw unedited blots and the uncropped blot with the relevant bands clearly labelled as Figure 4B. [file elife-83946-fig4-data1.zip › Figure 4B-source data 1-orignal.tif]

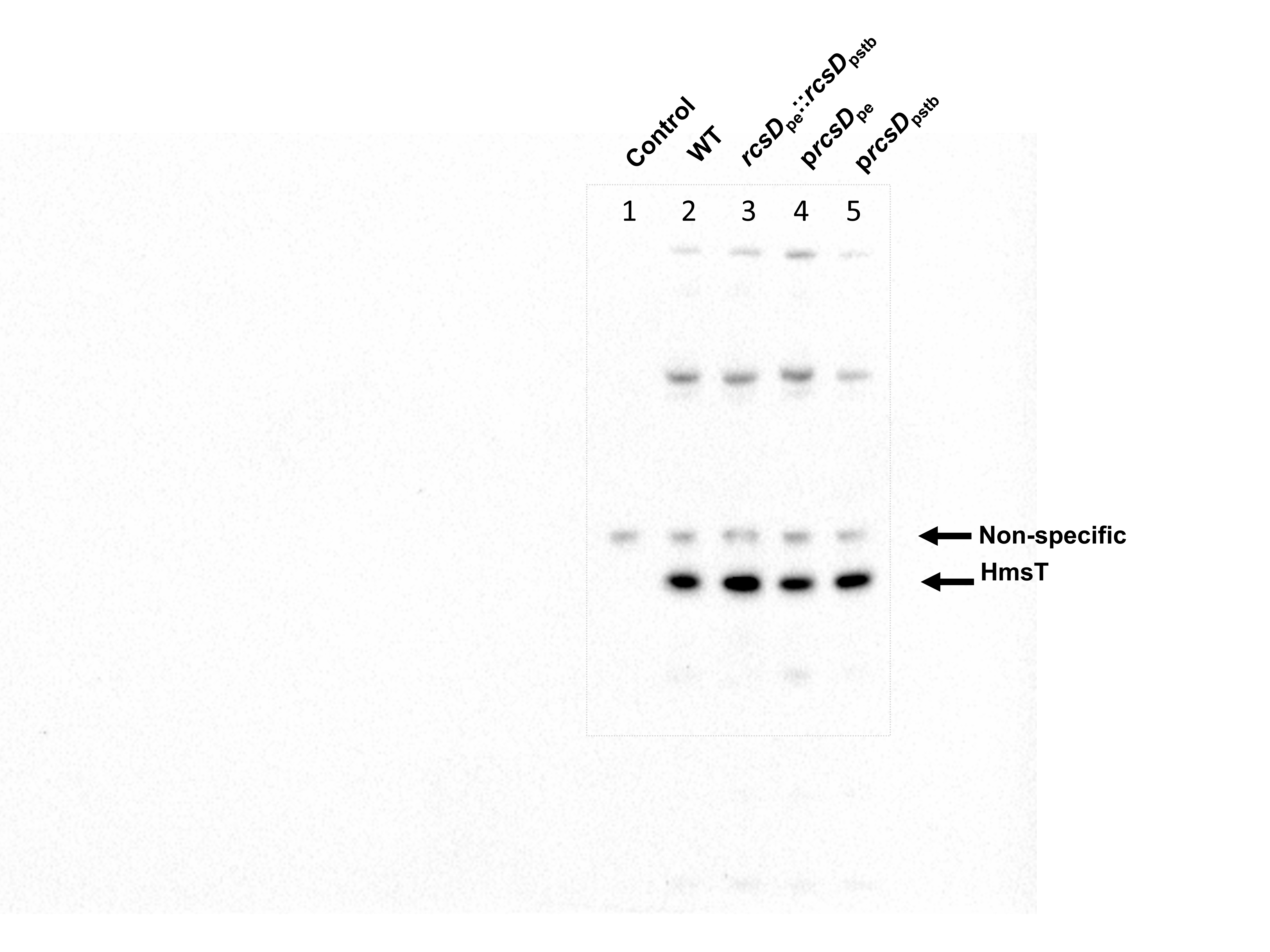

Supplement: Figure 4—source data 2. — The original file of the full raw unedited blots and the uncropped blot with the relevant bands clearly labelled as Figure 4D. [file elife-83946-fig4-data2.zip › Figure 4D-source data 2-labellede.TIF]

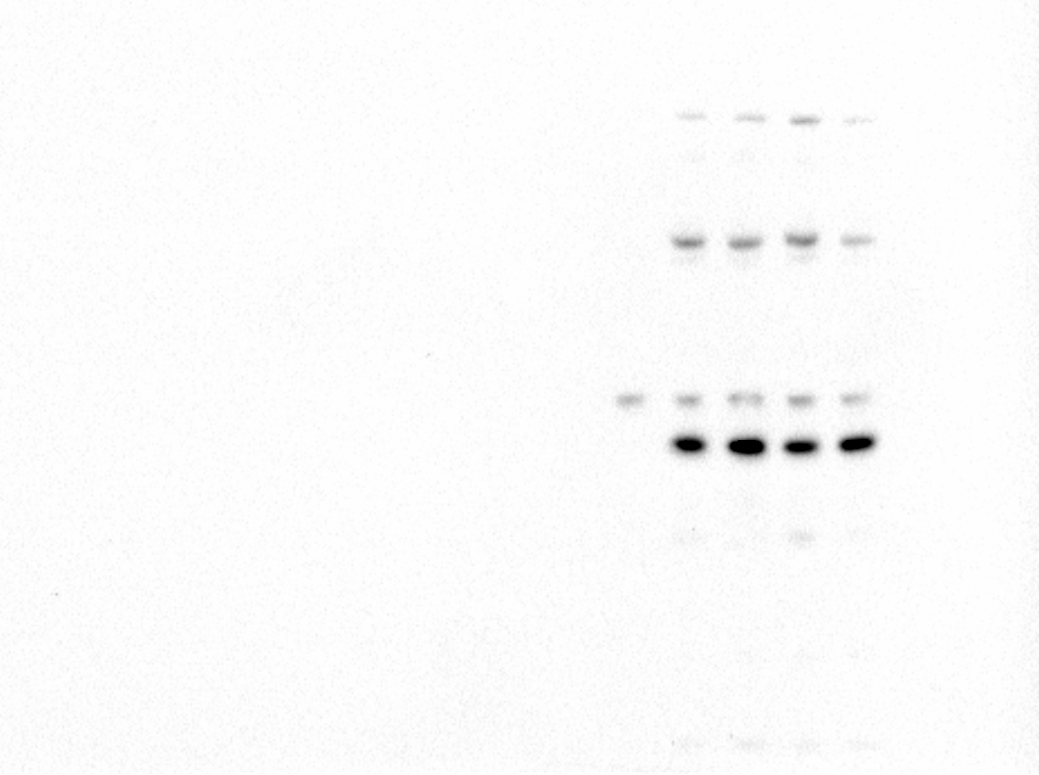

Supplement: Figure 4—source data 2. — The original file of the full raw unedited blots and the uncropped blot with the relevant bands clearly labelled as Figure 4D. [file elife-83946-fig4-data2.zip › Figure 4D-source data 2-orignal.tif]
