# Supplementary material for: A frameshift in Yersinia pestis rcsD alters canonical Rcs signalling to preserve flea-mammal plague transmission cycles
Source: eLife. 2023 Apr 3;12:e83946. doi: 10.7554/eLife.83946 (PMC10191623; doi:10.7554/eLife.83946)
Supplement: Supplementary file 1. [file elife-83946-supp1.docx]

## Supplementary File 1. Strains used in this study.

| **Strain** | **Genotype and description** | **Reference** |
| --- | --- | --- |
| ***Yersinia pestis* KIM6+** | | |
| WT | Wild type of KIM6+ | (Deng et al., 2002) |
| ∆*rcsD*_N-term_ | Fragment between 1 and 1846 bp of *rcsD* deleted in KIM6+ | (Sun et al., 2008) |
| *rcsD*_pe_::*rcsD*_pstb_ | *rcsD*_pe_ substituted with *rcsD*_pstb_ in KIM6+ | (Sun et al., 2008) |
| ∆*rcsD* | Fragment between 1 and 2530 bp of *rcsD* deleted in KIM6+ | This study |
| *∆rcsD*_C-term_ | Fragment between 1898 and 2530 bp of *rcsD* deleted in KIM6+ | This study |
| ∆*rcsB* | *rcsB* gene deleted in KIM6+ | (Sun et al., 2008) |
| *rcsD*_pe_ (H844A) | *rcsD*_pe_ mutated in KIM6+, at a site of conserved histindine of RcsD (RcsD^H844A^) | This study |
| ∆*rcsD*_N-term_ (H844A) | *rcsD*_pe_ mutated in ∆*rcsD*_N-term_, at a site of conserved histindine of RcsD (RcsD^H844A^) | This study |
| *rcsD-hpt* (RBS*) | The predicted RBS of *rcsD-hpt* mutated in KIM6+ (C)AAAAGG > (A)ACAAAG) | This study |
| *rcsD* (ATT^312^**🡪**GGT) | The predicted translation start codon of *rcsD-hpt* mutated in KIM6+（ATT > GGT) | This study |
| *rcsD* (ATT^-312^**🡪**ATG) | The predicted translation start codon of *rcsD-hpt* mutated in KIM6+（ATT > ATG) | This study |
| *rcsD*_pe_ (inserting stop codon before frameshift) | inserting stop codon before the 7T frameshift of *rcsD*_pe_ | This study |
| ∆*rcsC* | *rcsC* gene deleted in KIM6+ | This study |
| *rcsD*_pe_::*rcsD*_pstb_ ∆*rcsC* | *rcsC* gene deleted in *rcsD*_pe_::*rcsD*_pstb_ | This study |
| ∆*rcsF* | *rcsF* gene deleted in KIM6+ | This study |
| *rcsD*_pe_::*rcsD*_pstb_ ∆*rcsF* | *rcsF* gene deleted in *rcsD*_pe_::*rcsD*_pstb_ | This study |
| ∆*igaA* | *igaA* gene deleted in KIM6+ | This study |
| *rcsD*_pe_::*rcsD*_pstb_ ∆*igaA* | *igaA* gene deleted in *rcsD*_pe_::*rcsD*_pstb_ | This study |
| *rcsB* (D56Q) | *rcsD*_pe_ gene mutated in KIM6+, at a site of conserved aspartic acid of RcsB (RcsB^D56Q^) | This study |
| ***Yersinia pestis* biovar Microtus strain 201** | | |
| WT | Wild type of biovar Microtus strain 201 | (Song et al., 2004) |
| *rcsD*_pe_::*rcsD*_pstb_ | *rcsD*_pe_ substituted with *rcsD*_pstb_ in strain 201 | This study |
| ∆*rcsB* | *rcsB* gene deleted in strain 201 | This study |
