# Supplementary material for: A frameshift in Yersinia pestis rcsD alters canonical Rcs signalling to preserve flea-mammal plague transmission cycles
Source: eLife. 2023 Apr 3;12:e83946. doi: 10.7554/eLife.83946 (PMC10191623; doi:10.7554/eLife.83946)
Supplement: Supplementary file 2. [file elife-83946-supp2.docx]

## Supplementary File 2. Plasmids used in this study.

| **Plasmid** | **Description** | **References** |
| --- | --- | --- |
| pUC19 (p)^a^ | Cloning vector, ColE1 replicon , Amp^r^ | Lab stock |
| *prcsD_pe_* | *rcsD*_pe_ gene cloned in pUC19 | This study |
| p*rcsD*_pstb_ | *rcsD*_pstb_ gene cloned in pUC19 | (Sun et al., 2008) |
| p*rcsD*_pe_ (H844A) | *rcsD*_pe_ gene with mutated conserved Histidine (H844A) cloned in pUC19 | This study |
| p*rcsD*_C-term_ | Fragment between 1828 and 2693 bp of *rcsD*_pe_ cloned in pUC19 | This study |
| p*rcsD*_C-term_ (H844A) | The conserved histidine (H844A) mutated in p*rcsD*_C-term_ | This study |
| p*rcsD*_C-term (648bp)_, p*rcsD*_2046- 2693_ | Fragment between 2045 and 2693 bp of *rcsD*_pe_ cloned in pUC19 | This study |
| p*rcsD*_pe_ (TTG^-573^**🡪**CTT) | Point mutation at a site 573 bp upstream of stop codon in p*rcsD*_pe_ (TTG > CTT) | This study |
| p*rcsD*_pe_ (TTG^-462^**🡪**TTA) | Point mutation at a site 462 bp upstream of stop codon in p*rcsD*_pe_ (TTG > TTA) | This study |
| p*rcsD*_pe_ (TTG^-573^**🡪**CTT, TTG^-462^**🡪**TTA) | Points mutation at two sites 573 bp and 462 bp upstream of stop codon in p*rcsD*_pe_ (TTG > CTT, TTG > TTA) in p*rcsD*_pe_ | This study |
| p*rcsD*_pe_ (ATT^-312^**🡪**GGT) | Point mutation at the predicted initiate site, 312 bp upstream of stop codon in p*rcsD*_pe_ (ATT> GGT) | This study |
| p*rcsD*_pe_ (inserting stop codon before frameshift) | inserting stop codon before the 7T frameshift | This study |
| p*rcsC* | *rcsC* gene cloned in pUC19 | This study |
| p*rcsC* (H489A) | *rcsC* gene with mutated HisKA domain (H489A) cloned in pUC19 | This study |
| p*rcsC* (D885A) | *rcsC* gene with mutated REC domain (D885A) cloned in pUC19 | This study |
| p*rcsC* (T913A) | *rcsC* gene with mutated REC domain (T913A) cloned in pUC19 | This study |
| p*rcsC* (D885A, T913A) | *rcsC* gene with mutated REC domain (D885A, T913A) cloned in pUC19 | This study |
| p*rcsC* (H489A, D885A) | *rcsC* gene with HisKA and REC domains (H489A, D885A) cloned in pUC19 | This study |
| p*rcsF* | *rcsF* gene cloned in pUC19 | This study |
| p*rcsF* (C125S) | *rcsF* gene with mutated domain (C125S) cloned in pUC19 | This study |
| p*igaA* | *igaA* gene cloned in pUC19 | This study |
| p*igaA* (C413S) | *igaA* gene with mutated periplasmic domain domain (C413S) cloned in pUC19 | This study |
| p*rcsB*-Flag | *rcsB* gene with fused C-terminal Flag tag cloned in pUC19 | This study |
| p*rcsB* (D56Q)-Flag | *rcsB* gene with mutated REC domain (D56Q) and fused C-terminal Flag tag cloned in pUC19 | This study |
| p*rcsD*_pe_-Flag | C-terminal Flag tag fused in p*rcsD*_pe_ | This study |
| p*rcsD*_pe_-Flag-His | C-terminal Flag and hexahistidine tags fused in p*rcsD*_pe_ | This study |
| p*rcsD*_C-term_-Flag-His | C-terminal Flag and hexahistidine tags fused in p*rcsD*_C-term_ | This study |
| p*rcsD*_pstb_-Flag | C-terminal Flag tag fused in p*rcsD*_pstb_ | This study |
| pBAD/Myc-His A (pBAD) | Expression vector, Amp^r^ | Invitrogen |
| pBAD*rcsD-hpt*_573bp_ | Fragment between 2121 and 2693 bp of *rcsD*_pe_ cloned in pBAD | This study |
| pBAD*rcsD-hpt*_462bp_ | Fragment between 2230 and 2693 bp of *rcsD*_pe_ cloned in pBAD | This study |
| pBAD/Myc-His A’ (pBAD’) | modified pBAD/Myc-His A without ATG | This study |
| pBAD’*rcsD-hpt* | Fragment between 2382 and 2693 bp of *rcsD*_pe_ cloned in pBAD’ | This study |
| pBAD’ *rcsD-hpt* (H844A) | The conserved histidine (H844A) mutated in pBAD’*rcsD-hpt* | This study |
| pBAD’ *rcsD-hpt (*ATT^-312^**🡪**GGT) | The predicted translation start codon of *rcsD-hpt* mutated in pBAD’*rcsD-hpt (*ATT > GGT) | This study |
| hmsT::lacZ reporter | 350 bp of *hmsT* upstream sequence, together with the first seven codons of the ORF cloned in pGD926 | (Guo et al., 2015) |
| pMal-*rcsD_pstb_*-*lacZ* | The partial sequence of *rcsD*_pstb_ (159 bp) containing the 8T (frameshifted region) cloned into pMAl-*lacZ* | This study |
| pMal-*rcsD_pe_*-*lacZ* | The partial sequence of *rcsD*_pe_ (158 bp) containing the 7T (frameshifted region) cloned into pMAl-*lacZ* | This study |
| pMal-*rcsD_pe_*-stop-*lacZ* | An additional stop codon was introduced into the 158-bp *rcsD*_pe_ sequence of pMal-*rcsD_pe_*-*lacZ* | This study |
| pKD46 | λ red recombination vector for gene deletion | (Datsenko and Wanner, 2000) |
| pAC-crRNA-cm | vector for ligating crRNA and gene editing | (Yan et al., 2017) |
| pKD46-Cpf1-amp | pKD46 with Cpf1, for gene editing | (Yan et al., 2017) |

pUC19 (p)^a^, represented as p for short. For p-derived plasmids, the fragment between 400 bp upstream of 5’ UTR DNA region of target gene and 100 bp downstream of its 3’ UTR DNA region was cloned into pUC19.
