# Supplementary material for: A frameshift in Yersinia pestis rcsD alters canonical Rcs signalling to preserve flea-mammal plague transmission cycles
Source: eLife. 2023 Apr 3;12:e83946. doi: 10.7554/eLife.83946 (PMC10191623; doi:10.7554/eLife.83946)
Supplement: Supplementary file 3. [file elife-83946-supp3.docx]

## Supplementary File 3. Primers and oligos used in this study.

| **Primers** | **Sequences** | **Function** |
| --- | --- | --- |
| p*rcsC* F | gctctagagcagaagatgaacatgttgttggt | Plasmid construction |
| p*rcsC* R | GGAATTCATTAGCCAAGTGGATAAAGAACTGt |  |
| p*rcsD* F | CGGGATCCGCCGCCTAAACGGTGATCg |  |
| p*rcsD* R | GGAATTCCGACAACGTTTACCCATTCAATT |  |
| pBAD*rcsD-hpt*_573bp_ F | catgccatggtaTtgttaaattcatttggtgctaact |  |
| pBAD*rcsD-hpt*_573bp_ R | ggaattcttatgggttaccttcctgcaaca |  |
| pBAD*rcsD-hpt*_462bp_ F | catgccatggtattgctcgcggcggatg |  |
| p*rcsD*_C-term_ F | CGGGATCCGCGGGTATTTCTGATGAAGAGAT |  |
| p*rcsD*_N-term_ R | GGAATTCCTGGTTTTTAGTTGCCTCTCATAGAG |  |
| *rcsC* deletion F | Tcggttatcggtagtatattctgttgttaacacagcctgtctggcgattccggggatccgtcgacc | Gene deletion by Recombineering |
| *rcsC* deletion R | atttttgccccgctcgactctcaagcccagttttgctctgcaatacgtgtaggctggagctgcttcg |  |
| *rcsD* deletion F | gcgccagagccgtcacgcgctaaccaagggaatcggcattatcgtt attccggggatccgtcgacc |  |
| *rcsD*_2530bp_ down deletion R | tgtacggtttgcgaaagtgatgttaaatcactttgctgaagttcagtgtaggctggagctgcttcg |  |
| *rcsD*_1898bp_ up deletion F | tatgtaaccaattatgtaaaaaattaaacggccaattagagattc attccggggatccgtcgacc |  |
| *rcsF* deletion F | TTGCTTCAATAGCACAACTTAAATGAATAAGGCTGAGGAATTTTTattccggggatccgtcgacc |  |
| *rcsF* deletion R | ttagatgaaacgttcagtgctgaaccctgacacaccgcttgttga gtgtaggctggagctgcttcg |  |
| *igaA* deletion F | tttcatagcgttgacgtaaatttaaagcgctctgatggggatggg attccggggatccgtcgacc |  |
| *igaA* deletion R | cgtgcatctgctggcatgagcgatgatcaaccggagatgggtgaggtgtaggctggagctgcttcg |  |
| *rcsD* fused 3xFlag F | gattataaagatcatgacatcgactacaaggatgacgatgacaagtgaccAtgaacaaccttaatgtaattattgctgatgac | Overlap PCR for mutagenesis |
| *rcsD* fused 3xFlag R | gtcgatgtcatgatctttataatcaccgtcatggtctttgtagtCtgggttaccttcctgcaacagtctg |  |
| *rcsD* fused 3xFlag and His6 F | ATGACGATGACAAGCATCATCATCATCATCACTGACCATGAACA ACCTTAATGTAATTATTGCTg |  |
| *rcsD* fused 3xFlag and His6 R | aggttgttcaTggTCAGTGATGATGATGATGATGCTTGTCATCGTCATCCTTGTAGTCg |  |
| *rcsC* (H489A) F | gcgGAATTGCGCACACCGCTGTATGGCATTATCGG |  |
| *rcsC* (H489A) R | AGCGGTGTGCGCAATTCCGCACTAACGGTAGCCAGAAACATGGATTTTG |  |
| *rcsC* (D885A) F | GCGGTGAACATGCCAAATATGGATGGTTACCGTTT |  |
| *rcsC* (D885A) R | ATATTTGGCATGTTCACCGCGGTCAATACCATATCTACGGTATTAGTATTTAACGC |  |
| *rcsC* (T913A) F | GCGGCGAATGCTTTGGCTGAGGGAAAACAAC |  |
| *rcsC* (T913A) R | TCAGCCAAAGCATTCGCCGCTACACCAATAATCGGGAAATTGTGATTCAA |  |
| *rcsD* (H844A) F | GCGCGATTAAAAGGCGTATTTGCCATGCTGAACC |  |
| *rcsD* (H844A) R | AATACGCCTTTTAATCGCGCTACGGTTTGCGAAAGTGATGTTAAATCACTT |  |
| *rcsB* (D56Q) F | AATTACTGAACTCTCTATGCCAGGGGATAAGTATGGTGATGGCATCAC |  |
| *rcsB* (D56Q) R | AGAGTTCAGTAATTAGCACGTTGGCATCAAGTTTGGACAAATTGTTAATAAGC |  |
| *rcsB* fused 3xFlag F | GATTATAAAGATCATGACATCGACTACAAGGATGACGATGACAAGtgaGCATGCCTGCAGGTCGACTCTAGA |  |
| *rcsB* fused 3xFlag R | GTCGATGTCATGATCTTTATAATCACCGTCATGGTCTTTGTAGTCAAGCTTGGCGTAATCATGGTCATAGCTGTTTCCT |  |
| *rcsD (*TTG^-462^**🡪**TTA) F | ttactcgcggcggatgaaacggggtttca |  |
| *rcsD* (TTG^-462^**🡪**TTA) R | Gtttcatccgccgcgagtaataatgtgtaattatcataatgttgcggattatccgttattgta |  |
| *rcsD (*TTG^-573^**🡪**CTT) F | cttcTAAATTCATTTGGTGCTAACTGCATTCTCACCGATGAAC |  |
| *rcsD (*TTG^-573^**🡪**CTT) R | GCACCAAATGAatttagaagTATTGTAATGATCGAGCGTACT |  |
| i*gaA* (C413S) F | CAGGGTAATGGCATGagtTATGTCCCCCCCAACATTCAAAACACACG |  |
| *igaA* (C413S) R | GTTGGGGGGGACATAactCATGCCATTACCCTGAGCCTTCAGCATATCG |  |
| *rcsF* (C125S) F | tatcaacaagcggtgAgtcagggttcagcactgaacgtttcatctaaatga g |  |
| *rcsF* (C125S) R | cagtgctgaaccctgacTcaccgcttgttgatagcagccagggac |  |
| *rcsD*_pe_ (ATT^-312^**🡪**GGT) F | \| aaaggttgagtacGGtacagaagatgatataaacctctatgagag \| \| --- \| \|  \| |  |
| *rcsD*_pe_ (ATT^-312^**🡪**GGT) R | \| tatcatcttctgtaCCgtactcaaccttttgttgcgattgttc \| \| --- \| |  |
| *rcsD*_pe_ (inserting stop codon before frameshift) F | ctgacatgattttttTatgtaaccaattatgtaaaaaattaaacggccaattagagattcgca |  |
| *rcsD*_pe_ (inserting stop codon before frameshift) R | ataattggttacataAAAAAAtcaTGTCAGCCCCGAACCATGATTGTATCTGTCG |  |
| Modified pBAD’ F | TTTTTTGGGCTAACAGGAGGttggatcccGGGGGTTCTCATCATCATCATCATCATGGTATGGC | Overlap PCR for ATG deletion in pBAD/Myc-His A |
| Modified pBAD’ R | TGATGATGATGAGAACCCCCgggatccaaCCTCCTGTTAGCCCAAAAAACGGGTATGGAGAAACA |  |
| *rcsD* (H844) crRNA F | TgcaaaccgtacatcgattaaaaggcGT | Cas12a mediated  genome editing |
| *rcsD* (H844) crRNA R | gccttttaatcgatgtacggtttgcATC |  |
| *rcsD* (H844A) oligo | tgatttaacatcactttcgcaaaccgtaGCGcgattaaaaggcgtatttgccatgctga |  |
| *rcsB* (D56) crRNA F | TctgacctctctatgccaggggataaGT |  |
| *rcsB* (D56) crRNA R | ttatcccctggcatagagaggtcagATC |  |
| *rcsB* (D56Q) oligo | caaacttgatgccaacgtgctaattactcagctctctatgccaggggataagtatggtg |  |
| *rcsD-hpt* (RBS)crRNA F | TtgtaatgtactcaaccttttgttgcGT |  |
| *rcsD-hpt* (RBS)crRNA R | gcaacaaaaggttgagtacattacaATC |  |
| *rcsD-hpt (*RBS*) Oligo | tttatatcatcttctgtaatgtactcaacTTTGTTttgcgattgttcagataaaattcg |  |
| *rcsD*_C-term_ (ATT^-312^) crRNA F | Tag tacattacagaagatgatataaaGT |  |
| *rcsD*_C-term_ (ATT^-312^) crRNA R | tttatatcatcttctgtaatgtactATC |  |
| *rcsD*_C-term_ (ATT^-312^ **🡪**GGT) oligo | tgaacaatcgcaacaaaaggttgagtacggtacagaagatgatataaacctctatga |  |
| *rcsD* _(_ATT^-312^**🡪**ATG) oligo | tgaacaatcgcaacaaaaggttgagtacATGacagaagatgatataaacctctatga |  |
| *rcsD*_pe_ (7T) crRNA F | \| TggggctgacatttttttatgtaaccGT \| \| --- \| \|  \| |  |
| *rcsD*_pe_ (7T) crRNA R | ggttacataaaaaaatgtcagccccATC |  |
| *rcsD*_pe_ (inserting stop codon before frameshift) oligo | cagatacaatcatggttcggggctgacatgatttttttatgtaaccaattatgtaaaaa |  |
| *rcsD*-*lacZ* frameshift test F | AtgaCcatggcgggtatttctgatgaag | Construction of pMal-*rcsD*_pe_ /*rcsD*_pstb_-*lacZ* and pMal-*rcsD_pe_*-stop-*lacZ* |
| *rcsD*-*lacZ* frameshift test F | gtacagatcttctactttactgcgaatctctaattg |  |
| *rcsD*_pe_-stop-*lacZ* | gtacagatcttatgctactttactgcgaatctctaattg |  |
| 16sRNA F | TGGGAGTGGGTTGCAAAAGA | qPCR |
| 16sRNA R | GTTACGACTTCACCCCAGTCA |  |
| *ybtT* qPCR F | CGTTCAGAATGCAGATGCGG |  |
| *ybtT* qPCR R | GAAGCATCCGTATCGCCTGA |  |
| *osmY* qPCR F | ACGCTTTTCACGCCGTTTAC |  |
| *osmY* qPCR R | GGTGTCGTACAGCTATCGGG |  |
| *glnH* qPCR F | TCAGCAGTACGGTGTTGCAT |  |
| *glnH* qPCR R | TTCAGCGTAAGTGCCGTCTT |  |
| *tus* qPCR F | ACTGATGCTGACAGAACGCA |  |
| *tus* qPCR R | CGTGAGCAATGGAGGGAACT |  |
| *hmsT* qPCR F | TGCTATCATCGTCGCCCAAG |  |
| *hmsT* qPCR R | ATTTCTGCCCGTGTCGTTCT |  |
| *igaA* qPCR F | CCATCCGGCGACGATTTTTC |  |
| *igaA* qPCR R | CATTGTCACTCTGGGTCGCT |  |
| *lsrF* qPCR F | ATTGGCATCCCACAGCAGAA |  |
| *lsrF* qPCR R | TAGCCATGGTCAAAGGCCAG |  |
| *irp2* qPCR F | TGGTAGCGATCTTCAGGGGA |  |
| *irp2* qPCR R | ACCGACTTCTTCCAGCAAGG |  |
| *pgm*-test F (*hmsS*) | CGCCCCTGATTTTTACGG | PCR for testing the loss of *pgm* locus |
| *pgm*-test R (*hmsS*) | CATCCCTGGCGTAAATGG |  |
| *pgm*-test F (upstream of *pgm* region) | ctttagattgtatgtaggcaagaga |  |
| *pgm*-test R (downstream of *pgm* region ) | TGAGGCAGGTAGGTATCG |  |
| *pgm*-test F (*ybtT*) as qPCR | CGTTCAGAATGCAGATGCGG |  |
| *pgm*-test R (*ybtT*) as qPCR | GAAGCATCCGTATCGCCTGA |  |
