# Supplementary material for: A frameshift in Yersinia pestis rcsD alters canonical Rcs signalling to preserve flea-mammal plague transmission cycles
Source: eLife. 2023 Apr 3;12:e83946. doi: 10.7554/eLife.83946 (PMC10191623; doi:10.7554/eLife.83946)
Supplement: Supplementary file 5. [file elife-83946-supp5.docx]

## Supplementary File 5. Mutations in *Y. pestis* during evolution involved in this study.

| **Gene** | **ID** | **Mutation in *Y. pestis*** | **Effect** | **Occurrence** |
| --- | --- | --- | --- | --- |
| *rcsA* | YPTB2486  YPO2449 | 30-bp internal duplication  in ORF | Derepression of HmsT caused by insertion of 10 residuals tandem repeat | All *Y. pestis*, except for branch 0 strain Pestoides A (0.PE4b) |
| *rcsD*^b^ | YPO0919 | Indel (8T**🡪**7T) in *rcsD*_C-term_ | Early termination of RcsD and accumulation of a small HPt encoded by *rcsD*_C-term_ | Most modern strains, except for 620024, CMCC05009 (0.PE7), I-3086 and A-1825 |
| *rcsD*^c^ | YPO0919 | Rearrangement, *rcsD_N-_*_term_ and *rcsD*_C-term_ -*rcsB*-*rcsC* were separated | Early termination of RcsD and accumulation of a small Hpt encoded by *rcsD*_C-term_ | Nairobi (1. ANT); Anloga (0.PE3); Algeria3 (1.ORI) |
| *rcsD*^d^ | YPO0919 | Indels in *rcsD*_N-term_ | Early termination of RcsD and accumulation of a small HPt encoded by *rcsD*_C-term_ | I-3086 (0.PE4m), A- 1825 (2.MED1) |
| IS*_ompC_* | YPO1900,  YPO1998 | Acquist of IS elements in *ompC* | Easy loss of *pgm* locus | Without IS: ancient, 91001, etc. (0.PE4;5; 2.MED);  With IS: CO92, KIM6+, etc |
| *igaA* | YPO0142 | Indels | Pseudogene | C-627, etc (0.PE4, 0.PE10, 0.PE5, 0.PE2, 2.MED) |
| *rcsC* | YPO0921 | Indel (8T**🡪**9T) | Pseudogene | Several strains (2.MED) |
| *rcsF* | YPO1070 | Indel | Pseudogene | D106004 (1.IN) |

*rcsD*^a^, *rcsD*^b^, and *rcsD*^c^ stand for three different mutations of *rcsD* in *Y. pestis* during evolution as described in this Table.
