# Supplementary material for: A frameshift in Yersinia pestis rcsD alters canonical Rcs signalling to preserve flea-mammal plague transmission cycles
Source: eLife. 2023 Apr 3;12:e83946. doi: 10.7554/eLife.83946 (PMC10191623; doi:10.7554/eLife.83946)
Supplement: Supplementary file 6. [file elife-83946-supp6.docx]

## Supplementary File 6. Putative SD and start codon sites in *rcsD* in Enterobacteriaceae.

| Species | Predicted SD^a^ | Spacer length between SD and start site | Predicted start site (fragment length of predicted *rcsD-hpt*) |
| --- | --- | --- | --- |
| *Yersinia pestis* | AAAAGG | 8 bp | ATT (312bp) |
| *Yersinia pseudotuberculosis* | AAAAGG | 8 bp | ATT (312bp) |
| *Escherichia coli* | AGGAAG | 14 bp | ATT (327 bp) |
| *Shigella boydii* | AGGAAG | 14 bp | ATT (327 bp) |
| *Shigella boydii* | GAGCAA | 4 bp | ATG (351 bp) |
| *Serratia fonticola* | AGAACA | 4 bp | ATG (357 bp) |
| *Proteus mirabilis* | GAGGC | 10 bp | TTG (372 bp) |
| *Yersinia frederiksenii* | AGGAAG | 7 bp | ATT (381 bp) |
| *Erwinia amylovora* | AGGACG | 8 bp | ATC (576 bp) |
| *Klebsiella quasipneumoniae* | AGGAGG | 14 bp | ATT (327 bp) |
| *Klebsiella quasipneumoniae* | AGCACG | 3 bp | ATG (351 bp) |

SD^a^, Shine–Dalgarno sequence.
